# Key mechanisms of angiogenesis in the infarct core: association of macrophage infiltration with venogenesis

**DOI:** 10.1186/s13041-025-01182-1

**Published:** 2025-02-14

**Authors:** Luping Xue, Wei Ouyang, Peiyun Qi, Yan Zhu, Xiaoru Qi, Xiao Zhang, Xiangjian Zhang, Lina Wang, Lili Cui

**Affiliations:** 1 Department of Neurology, The Second Hospital of Hebei Medical University, Shijiazhuang, Hebei 050000 China; 2 Hebei Collaborative Innovation Center of Cardio-cerebral Vascular Disease, Shijiazhuang, Hebei 050000 China; 3 Hebei Key Laboratory of Vascular Homeostasis, Shijiazhuang, Hebei 050000 China; 4 Core Facilities and Centers, Hebei Medical University, Shijiazhuang, Hebei 050000 China; 5 Thyropathy Hospital, SUN Simiao Hospital of Beijing University of Chinese Medicine, Tongchuan Hospital of Traditional Chinese Medicine), Tongchuan, Shaanxi 727031 China; 6 Interventional Department of Cerebrovascular Disease, Cangzhou People’s Hospital, Cangzhou, Hebei 061000 China

**Keywords:** Cerebral ischemia, Angiogenesis, Venogenesis, Identification of arteries and veins, GIAO staining, Macrophage

## Abstract

Angiogenesis in the ischemic penumbra compensates for microcirculatory dysfunction and promotes neuronal plasticity after stroke. However, the current understanding may be highly biased because the contribution of veins to angiogenesis has been overlooked. This study revealed that the remodeling processes of veins differ from those of arteries after ischemia. Ligation of the right jugular vein increased the infarct volume, decreased cerebral blood flow and impaired long-term functional restoration after stroke. RNA-seq analysis revealed significant upregulation of the expression of genes associated with angiogenesis in the infarct core during the recovery period. By using gelatin ink-alkaline phosphatase-oil red O (GIAO) staining, we found that venogenesis, the process of creating new veins, was the predominant angiogenic event in the infarct core. Macrophage infiltration and transformation are closely associated with venogenesis in the infarct core. However, depletion of macrophages in the circulation by clodronate liposomes in the acute phase inhibited the proliferation of endothelial progenitor cells and decreased the vascular density in the infarct core. This study demonstrated that dynamic vein remodeling is crucial for cerebral ischemic damage and subsequent neuronal restoration. Angiogenesis occurs in the infarct core during the recovery period, promotes the absorption of necrotic tissue and facilitates functional recovery after stroke.

**Supplementary Information:**

The online version contains supplementary material available at 10.1186/s13041-025-01182-1.

## Introduction

Other than extending the window for intravenous thrombolysis and endovascular therapy, no other significant advancements have been made in the stroke field in recent decades [1]. Neurologists have recently realized that the development and clinical prognosis of cerebral infarction may be significantly influenced by the cerebral venous system. The production of microemboli, nonreflow phenomena after reperfusion, and arterial collateral capacity in the acute stage of cerebral infarction have all been documented to be related to venous drainage [2]. Favorable venous outflow profiles predict effective first-pass reperfusion after endovascular thrombectomy [3]. However, the mechanism underlying cerebral vein remodeling during brain repair following ischemia is still unknown because of the absence of experimental techniques to distinguish venules from arterioles.

Angiogenesis in the ischemic region promotes brain repair by increasing metabolism in surviving neurons in the acute phase and is a therapeutic target for promoting functional recovery after stroke [4–6]. Two weeks after stroke, the microvascular system in the ischemic brain exhibits a complex sinusoid structure and a high level of vascular proliferation [7]. An increased microvessel density in the peri-infarct region has been demonstrated to be associated with longer survival in stroke patients [8]. Additionally, angiogenesis might also be a key element in neuronal plasticity, as it increases blood flow and provides nutrients to damaged brain areas [5]. Increasing evidence has demonstrated that microvessel remodeling is related to axonal growth, synaptic remodeling, and functional remapping after stroke. Many elements, such as neurotrophins, growth factors, adhesion molecules, and progenitor cells, which participate in the process of angiogenesis, also contribute to brain remodeling through intricate pathways. However, the current understanding may be highly biased because the contribution of veins to angiogenesis has been overlooked.

The ischemic penumbra is a flexible region with high potential for vascular and neuronal plasticity [5, 9]. Revascularization in the peri-infarct region alleviates ischemic damage and promotes functional restoration by increasing cerebral blood flow (CBF) and creating pathways for the migration of neural progenitor cells [10, 11]. In 2019, Kanazawa et al. [9] proposed that the function and outcomes of angiogenesis in the ischemic core and penumbra may differ. Previous studies have focused mainly on evaluating the role of angiogenesis in the ischemic penumbra in brain repair. However, vascular alterations in the infarct core following stroke have not been reported. However, whether angiogenesis occurs and how vascular remodeling in the infarct core affects functional recovery after stroke are still unknown. In this study, we developed a histochemical method to distinguish venules from arterioles in the brain parenchyma microvessel network and explored the effects of angiogenesis in the infarct core on functional recovery following stroke.

## Materials and methods

### Animals and focal cortical infarction

Male C57BL/6 mice (12–16 weeks, 25 ~ 30 g; River Laboratory Animal Technology Company) were utilized for the study. Cerebral cortex infarction was induced by permanently cauterizing the middle cerebral artery (MCA) under a visible skull window via a previously reported method [12].

### Infarct volume assessment

2,3,5-Triphenyltetrazolium chloride (TTC) staining was employed to measure the cerebral infarct volume. The mouse brains were coronally sliced into 1.5 mm thick sections, immersed in 2% TTC, and then incubated at 37 °C for 10 min. After postfixation in 4% paraformaldehyde for 24 h, the brain sections were imaged under a macro-optical zoom microscope (Axio Zoom. V16, Zeiss).

### Neurobehavioral assessment

Before the surgery, the mice underwent a two-day training session on a rotarod at a constant pace. Motor coordination post-MCA occlusion (MCAO) was subsequently assessed via an accelerating rotarod from RWD Life Science Co. in China. The initial speed was set at 4 rpm and was gradually increased to 40 rpm over 5 min. The duration for which the mice remained on the rod was recorded as the falling latency. Three trials were conducted each day, and the average falling latency was analyzed.

### Gelatin-ink perfusion for quantification of microvessel density

Vessel density was measured in brain sections, in which blood vessels were labeled with a black ink-gelatin mixture. A thermostatic water bath heated to 55 °C was used to dissolve the gelatin (Sigma‒Aldrich, USA) to a 4% concentration in normal saline. The mixture composed of 4% gelatin and 10% black Chinese painting ink (Yi De Ge, China) was then filtered. The mice were subsequently perfused with preheated saline and 4% paraformaldehyde. A total of 100 ml of the gelatin-ink mixture warmed in a 65 °C water bath was pumped into the heart at a rate of 10 ml/min. The mice were subsequently placed on the ventral side at 4 °C overnight. Thereafter, the brain samples were postfixed in 4% paraformaldehyde solution and dehydrated in 30% sucrose. Serial frozen sections with a thickness of 100 μm were extracted and mounted on slides using glycerol gelatin mounting medium for microscopy. The vessel density was measured as the percentage area of microvessels via ImageJ software.

### Gelatin ink-alkaline phosphatase-oil red O (GIAO) staining

We developed GIAO staining to distinguish the arterial and venous segments of capillaries in gelatin-ink labeled brain slices [12]. This approach was based on the restricted localization of alkaline phosphatase (ALP) on arterial capillaries but not veins and was modified from a protocol published in 1988 by Ushiki and Abe [13]. Red Chinese ink was infused into the mice to label vessel lumens via the above method. ALP staining was then performed to label arteries on 15 μm brain slices according to the protocol from Solarbio Science & Technology Co., China. The sections were incubated with a combination of FBB and naphthol AS-BI phosphate for 15 min, washed in distilled water for 5 min, and then counterstained with nuclear fast red or methyl green for 3 min. Afterward, the sections were thoroughly rinsed with PBS to remove excess dye. To further enhance the contrast of the red ink color, the sections were counterstained with oil red O (Solarbio Science & Technology Co., China) for 10 min in the presence of 60% isopropyl. This additional step helped to visualize the bright red lumens of the microvessels under a light microscope (Zeiss Axio scope A1) following the oil red O staining process.

### Immunofluorescence staining

The brain sections were permeabilized with 0.3% Triton X-100 for 30 min and blocked with 10% normal donkey serum. The following primary antibodies were used to incubate the brain sections overnight at 4 °C: sheep polyclonal anti-BrdU (Abcam), purified rat anti-mouse CD31 (BD Pharmingen™), 488-conjugated Arginase-1 polyclonal (Proteintech), 488 rabbit anti-mouse CD68 (ABclonal Technology), and CD206 monoclonal (MR5D3) (Invitrogen) antibodies. The sections were subsequently incubated with appropriate secondary antibodies (Alexa Fluor 488 and 594; Jackson ImmunoResearch, USA) at 37 °C for 1 h. To detect the proliferation of endothelial cells, BrdU was administered by intraperitoneal injection at a dose of 50 mg/kg for 14 days. To quantify the blood vessel density in the infarct core, the CD31-positive area was measured in two brain slices from each mouse. Five to eight image fields (200×) in the infarct core were obtained for each brain slice.

### Transmission electron microscopy (TEM)

The mice were transcardially perfused with 4% glutaraldehyde. The brain samples containing the motor cortex were then cut into 1 mm^3^ pieces, dehydrated in graded acetone solutions, and embedded in Epon 812. Ultrathin sections were taken from representative brain regions, such as the peri-infarct cortex, infarct core, and healthy cortex. Ultrathin Sect. (50 nm) were mounted onto 150 mesh copper grids, stained with lead citrate and uranium acetate, and then examined via TEM (HT7800, Japan).

### Thinned-skull cranial window

The mice were anesthetized and then fixed on a stereotaxic instrument (RWD Life Science Co., China). After the skin above the skull was disinfected and the skull was exposed, the skull was wiped clean with wet cotton swabs. Then, we outlined a 5-mm-diameter region of interest (ROI) on the skull over the ischemic cortex and removed the outer layer of compact bone and most of the spongy bone using a high-speed microdrill. Normal saline was used to moisten and cool the skull to prevent thermal injury caused by the high-speed drill. After removing most of the spongy bone, we artificially thinned the inner layers of compact bone with a surgical blade until the meningeal vessels were clearly visible under a microscope.

### Laser speckle contrast imaging (LSCI)

CBF was measured before, 24 h, 48 h and 72 h after cerebral ischemia using LSCI (PeriCam PSI system, Sweden). The surface of the intact mouse skull was illuminated by a 785 nm laser at an acquisition rate of 5 images per second. A laser head coupled with a camera was positioned 15.0 ± 5.0 cm above the skull. The perfusion scale of the ROI was determined automatically with an intensity filter of 5 to 10.00.

### Live imaging of meningeal vessels in the peri-infarct region

The leptomeningeal vessels were imaged through the thinned-skull window using a zoom stereo microscope (Zeiss Axio Zoom V16, Germany). The end of a plastic pipette (5 ml) was trimmed to create a customized water reservoir and subsequently attached to the ROI on the skull. To enhance image resolution, the container was filled with an appropriate quantity of normal saline. Zeiss analysis software was used to measure the length and diameter of the draining veins in the ROI. We measured both the length and straight-line distance of the veins. The tortuosity of the veins was determined by calculating the ratio of vessel length to straight-line distance. The veins, MCA branches, and vein branch terminals were outlined via Photoshop 2020, and the veins and arteries were pseudocolored with different colors. The terminal size was subsequently measured using ImageJ.

### Macrophage depletion

Macrophages in the blood circulation are depleted by intravenous injection of clodronate-loaded liposomes [14]. A significant reduction in macrophages can typically be achieved within 24–48 h with a single dose of clodronate-liposome injection, which can last for 4–5 days. On day 4 after MCAO, the mice were anesthetized with isoflurane. The liposome tube was inverted several times until the mixture was homogenized. A total volume of 250 µL of clodronate liposomes or control liposomes was injected via the retro-orbital venous sinus via a 1 mL syringe. On day 8 after MCAO, the mice were transcardially perfused and fixed in paraformaldehyde for subsequent ALP and CD31 staining.

### RNA sequencing (RNA-seq) and data analysis

Samples of the infarct core, control cortex, and peri-infarct cortex were collected 10 days after ischemic stroke for RNA-seq analysis. The peri-infarct tissue was specifically defined as the 2 mm cortex surrounding the infarct core. To separate the peri-infarct cortex from the infarct core, a surgical blade was used to cut along the visible border of the necrotic tissue, which appeared pale in color. Total RNA was extracted using TRIzol reagent (Solarbio). The NEBNext Ultra RNA Library Prep Kit for Illumina (NEB) was used to construct sequencing libraries. The PCR products were subsequently purified, and the library quality was evaluated on an Agilent Fragment Analyzer 5400 system. Index-coded samples were clustered on a cBot cluster generation system using the TruSeq PE Cluster Kit v3-cBot-HS (Illumina). The RNA libraries were then sequenced on an Illumina NovaSeq 6000 platform. High-quality paired-end clean reads were aligned to the mouse reference genome using HISAT2 v2.2.1. The FPKM values of genes were calculated to obtain mRNA expression profiles. Differential expression analysis for samples with biological replicates was carried out with the DESeq2 R package (1.26.0) to identify differentially expressed mRNAs (fold change ≥ 2 and p value < 0.05). Gene Ontology (GO) enrichment analysis of the differentially expressed genes was performed using DAVID (2021). We used KOBAS v3.0 software to assess the significant enrichment of the differentially expressed genes (DEGs) in different KEGG pathways. KEGG pathways with P values less than 0.05 were considered significantly enriched for the DEGs. All RNA-seq data were deposited in the GEO database (GSE138805).

### Statistical analysis

SPSS version 19.0 software was used to perform the statistical analysis. *p* ≤ 0.05 was considered to indicate statistical significance. All the data are presented as the means ± standard errors of the means (SEMs). The significance of differences among the three groups was determined by one-way ANOVA followed by least significant difference (LSD) correction. Unpaired Student’s t test or paired sample t test was used to analyze differences between two groups. Statistical charts were generated with GraphPad Prism software (v 9.00). Enrichment analysis charts were plotted with http://www.bioinformatics.com.cn, a free online platform for data analysis and visualization.

## Results

### Dynamic vein remodeling in the peri-infarct region is associated with ischemic injury and recovery

**Fig. 1:**
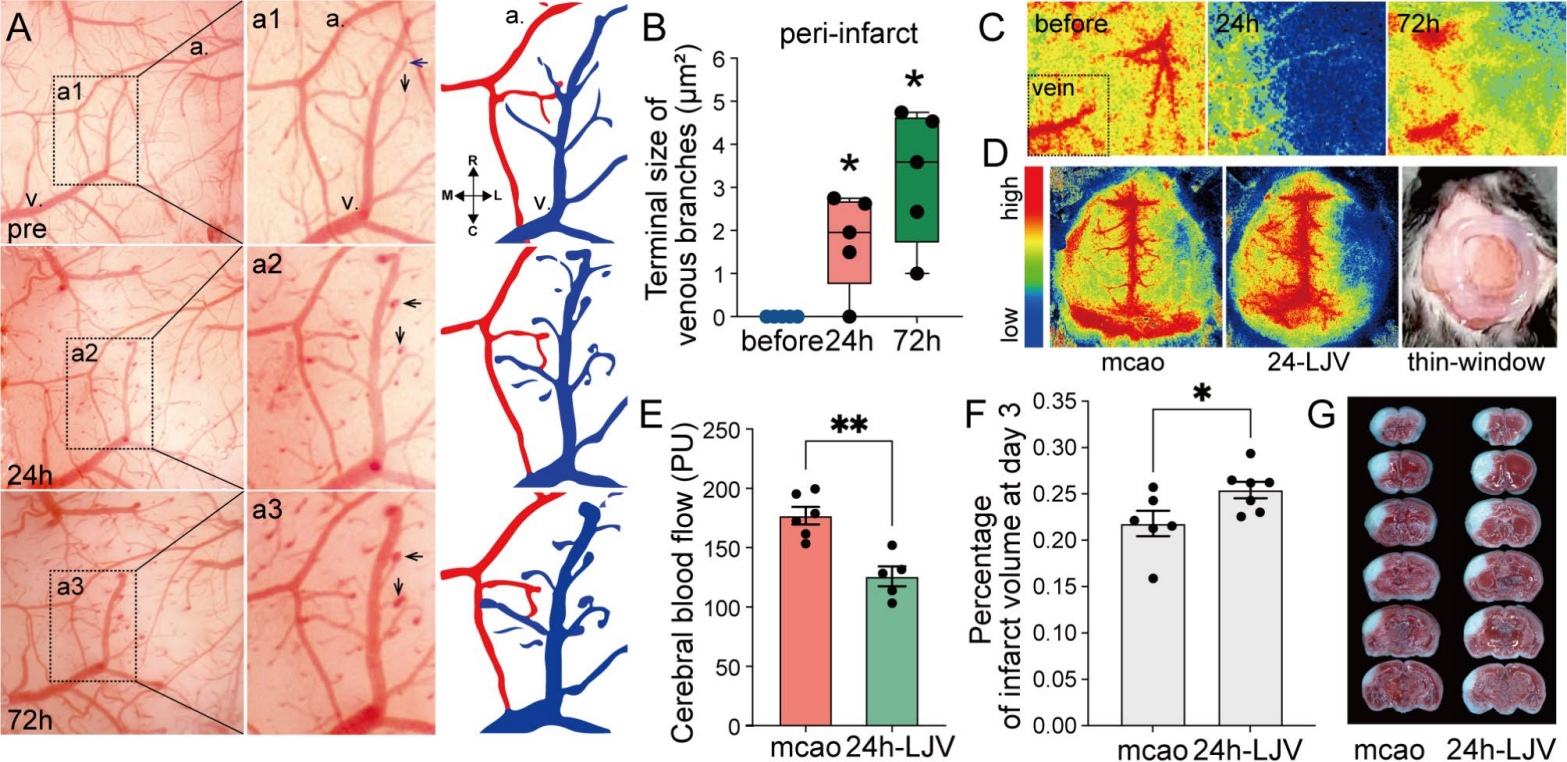
Structural changes in veins were associated with ischemic injury in the acute phase. (**A**) Sequential live imaging revealed that the terminals of vein branches were enlarged in the peri-infarct region in the acute phase. Arrows, enlarged terminals. The arteries and veins selected in A were pseudocoloured. Blue and v., veins. Red and a., arteries. (**B**) Statistical charts showing an increase in the size of the terminal in the acute phase. *n* = 5, before vs. 24 h, *p* = 0.026; before vs. 72 h, *p* = 0.001. (**C**) LSCI under a thinned-skull window was used to detect cerebral blood flow (CBF) changes in the same mice as shown in graph A. (**D**), (**E**) and (**F**) Ligation of the right jugular vein at 24 h (24 h-LJV) decreased the ipsilateral CBF (*n* = 5 ~ 6, *p* = 0.0013) and increased the infarct volume 3 days later (*n* = 6, *p* = 0.0433). (**G**) TTC staining showing the infarct size on day 3 postischemia

By creating a thinned-skull window, we observed that the remodeling processes of veins differ from those of arteries after ischemia (Fig. 1). The veins were elongated and enlarged at 24 and 72 h after ischemia, with no new branches forming (Fig. 1A). The venous branch terminals in the peri-infarct region became enlarged at 24 h and grew significantly larger at 72 h (Fig. 1A-C). Ligation of the right jugular vein at 24 h (24 h-LJV) significantly decreased the ipsilateral CBF and increased the infarct volume (Fig. 1D-G).

**Fig. 2:**
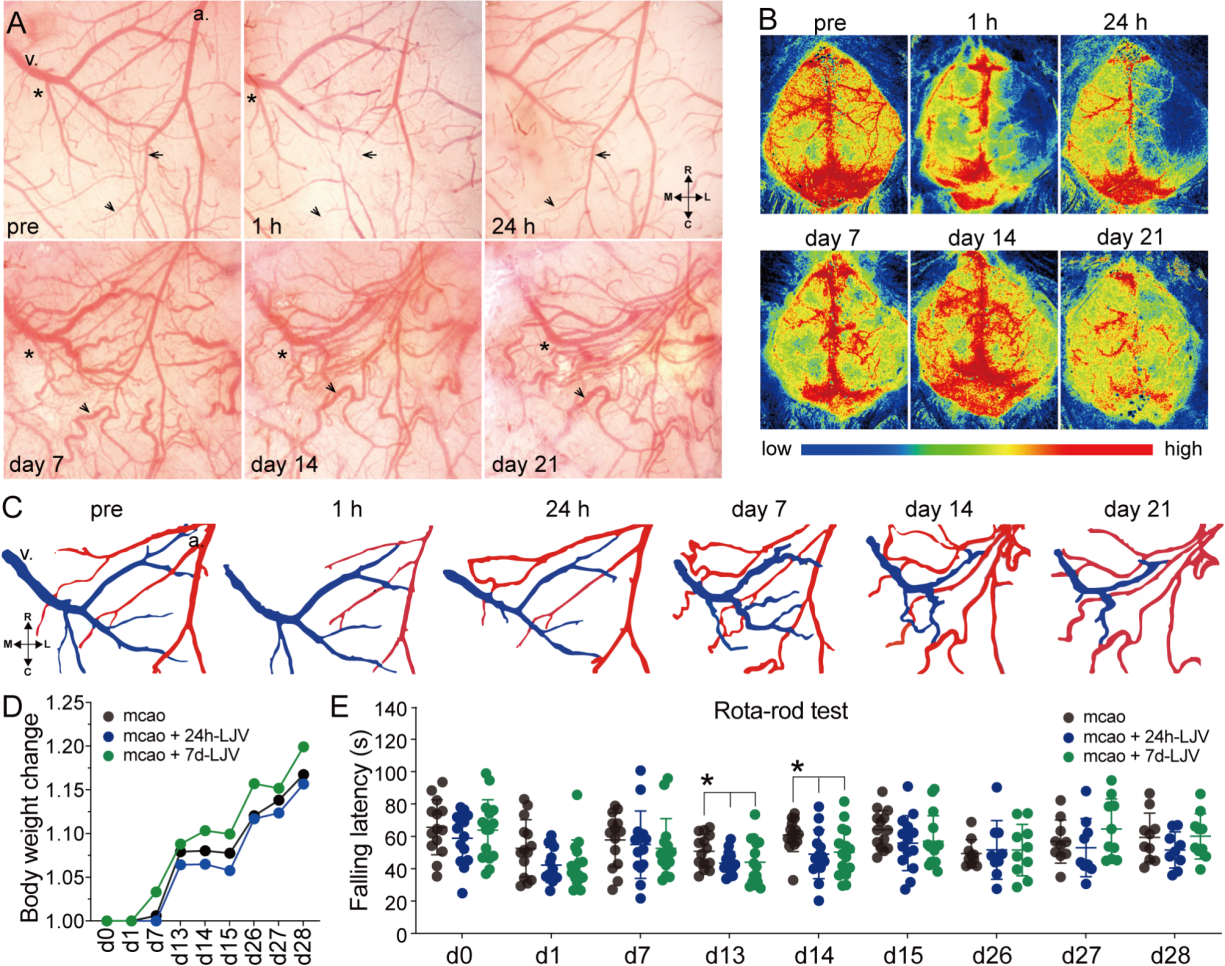
Dynamic remodeling of veins during the chronic stage was associated with functional restoration. **(A)** and **(B)** Long-term live images and LSCI images showing dynamic vascular remodeling and CBF changes in the peri-infarct region. The arrow indicates that the arterial branch was constricted at the onset of ischemia and subsequently refilled within 24 h. a., artery. v., vein. Arrow head, arterial collateral formation. Asterisk, dynamic vein remodeling. **(C)** Pseudocoloured vessels selected from (A) showing the remodeling of arteries and veins after ischemia. Blue, veins. Red, arteries. **(D)** Ligation of the right jugular vein did not affect the ratio change of mice weight after stroke. mcao, middle cerebral artery occlusion. 7d-LJV, ligation of the right jugular vein on day 7. **(E)** Mice in both the 24 h-LJV and 7d-LJV groups presented a decreased latency to fall on the rotarod at week 2, suggesting that ligation of the right jugular vein either at 24 h or at 7 days impaired the recovery of motor function after infarction. *n* = 10 ~ 15. *p < 0.05. d13: mcao vs. 24 h-LJV, *p* = 0.037; mcao vs. 7d-LJV, *p* = 0.045. d14: mcao vs. 24 h-LJV, *p* = 0.029; mcao vs. 7d-LJV, *p* = 0.047

Seven days later, the curvature of the venous branches increased along with elongation of the collateral artery and absorption of necrotic tissue (Fig. 2A-C). New collateral anastomoses were established and gradually served as the main supplying artery to the ischemic region, while the vein branches retracted following necrotic tissue absorption after 14 days (Fig. 2C). To further explore the effects of vein remodeling on ischemic injury and neuronal function after stroke, we assessed mouse motor ability following ligation of the right jugular vein. Ligation of the right jugular vein did not significantly affect the changes in the ratio of mice weight following ischemia (Fig. 2D). At week two, the falling latency of the mice in both the 24 h-LJV and 7d-LJV (ligation of the right jugular vein at 7 days) groups was significantly shorter than that of the control group (Fig. 2E). These findings indicate that blockage of venous drainage in the acute or recovery stage impaired functional recovery.

### Dynamic vein remodeling in the peri-infarct region is associated with ischemic injury and recovery

**Fig. 3:**
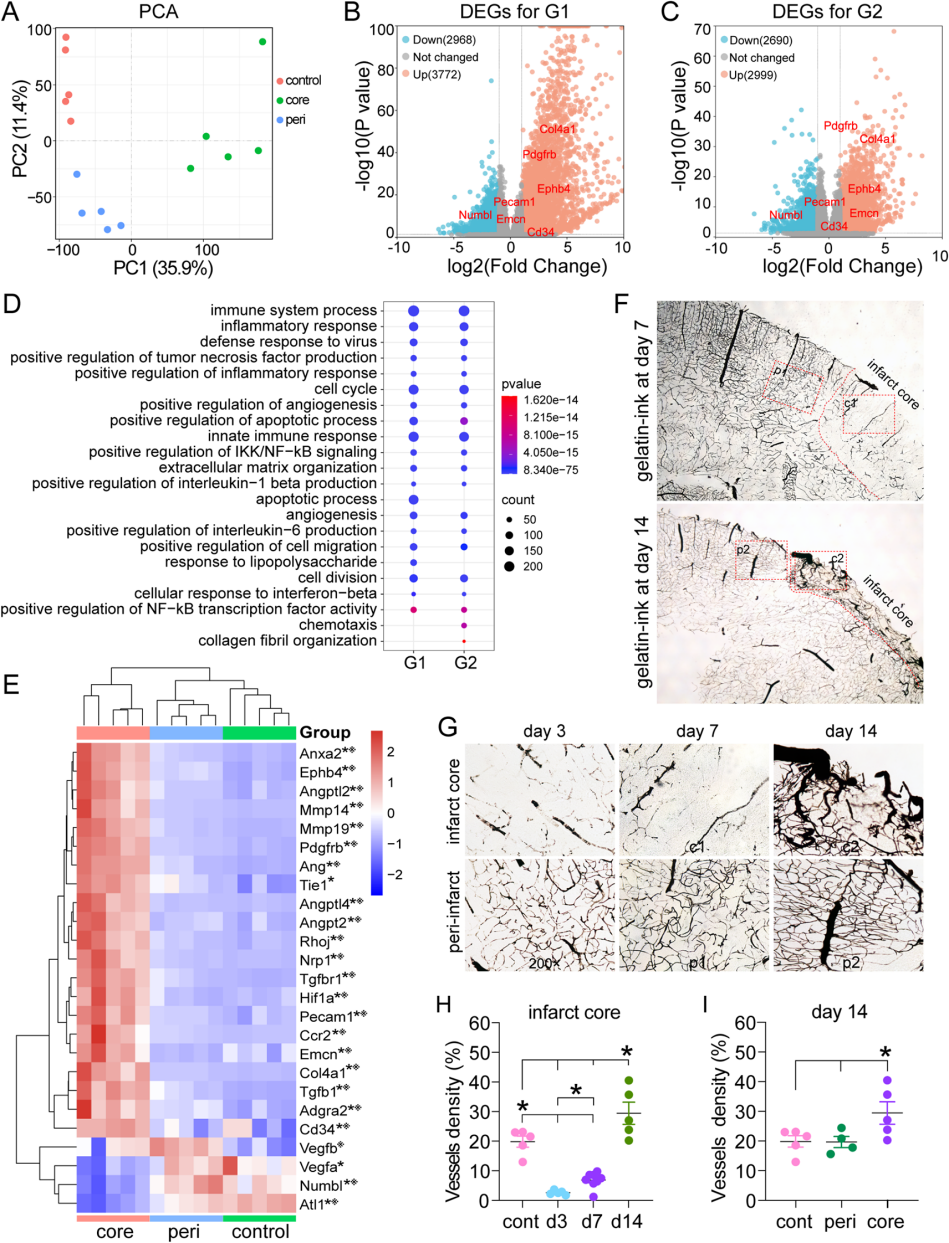
The infarct core region displayed substantial angiogenic activity during the recovery period. **(A)** ~ **(E)** RNA-seq analysis of differentially expressed genes (DEGs) on day 10. *n* = 5. **(A)** PCA revealed distinct clustering of genes in control cortical tissue, the peri-infarct cortex and the infarct core. **(B)** and **(C)** Volcano plot showing the DEGs with p values < 0.05 for the G1 and G2 comparisons. G1, control vs. core. G2, peri vs. core. **(D)** The top 20 enriched GO terms for the upregulated genes revealed that angiogenesis was significantly enriched in the infarct core compared with the control cortex and peri-infarct cortex. **(E)** Heatmaps showing the gene expression of angiogenesis-associated factors. *p < 0.05, infarct core (core) vs. control cortex. ^※^*p* < 0.05, infarct core vs. peri-infarct cortex (peri). **(F)** ~ **(G)** Brain sections labeled with gelatin-ink on days 3, 7 and 14. Middle cerebral artery occlusion impaired the microvascular structure in the infarct core on days 3 and 7. The microvessels proliferated in the peri-infarct and infarct cores on day 14. **(H)** Statistical analysis revealed that the vessel density in the infarct core was significantly increased on day 14 and had increased since day 7. *n* = 5 ~ 7. *p < 0.05. d3 and d7 vs. control, *p* < 0.0001. d14 vs. d3 and d7, *p* < 0.0001. d14 vs. cont, *p* = 0.004. **(I)** The infarct core had a greater vessel density than the control cortex on day 14 after ischemia. *n* = 5 ~ 7. core vs. control, *p* = 0.028. core vs. peri, *p* = 0.034

Principal component analysis (PCA) revealed distinct clustering of mRNAs for control cortical tissue, peri-infarct cortex, and infarct core samples on day 10 after ischemia (Fig. 3A), indicating that gene expression patterns were significantly different between these tissues. We detected 6,740 DEGs, including 3,772 upregulated genes and 2,968 downregulated genes, in the infarct core compared with those in the control cortical tissue (Fig. 3B. G1: comparison between the normal control cortex and infarct core). A total of 5,689 DEGs, including 2,999 upregulated genes and 2,690 downregulated genes, were identified in the infarct core compared with the peri-infarct cortex (Fig. 3C; G2: comparison between the peri-infarct cortex and infarct core). The key downregulated genes were involved mainly in neuronal synapses and ion channel activity. Among these DEGs, a total of 2454 (fold change ≥ 2 and *p* < 0.05) were simultaneously upregulated in the infarct core compared with the control cortical tissue or the peri-infarct region. GO annotation of these upregulated mRNAs revealed that they were enriched mainly in biological processes such as immune, inflammatory and apoptotic processes (Fig. 3D). Unexpectedly, GO analysis revealed that there were 101 upregulated DEGs in the infarct core that were associated with angiogenesis, including proangiogenic genes (Ephb4, Angpt, Pdgfrb, Tie1, Tgfbr1, Hif1a, Ang, MMP, etc.), and vascular endothelial cell markers (Pecam1, Col4a1, and Cd34) (Fig. 3D and E; Fig. S1A). These factors play significant roles in the regulation of blood vessel morphogenesis, blood vessel development and remodeling, and sprouting angiogenesis (Fig. S1B). These results indicate that, in addition to inflammation and apoptosis, angiogenic activity may be increased in the infarct core compared with the peri-infarct region during the recovery phase after stroke.

### Microvessel density significantly increased in the infarct core region during the recovery period following ischemia

The gelatin-ink perfusion method (Fig. 3F-I) was then used to detect vessel density in the infarct core in 100 μm thick brain sections. Vascular remodeling in the peri-infarct area occurred predominantly at week 2 after ischemia, as evidenced by a significant increase in the peri-infarct vessel density (VD-peri) on day 14 and radial expansion of the peri-infarct microvessels toward the infarct core (Fig. 3F and G). Very few microvessels filled with gelatin-ink were observed in the infarct core on day 3 as a result of MCA occlusion. On day 7, a small proportion of discontinuous microvessels were visible in the infarct core, making up 6.87 ± 2.64% of the visual field, a slight increase compared with that observed on day 3 (2.64 ± 0.74%) (Fig. 3F, H). On day 14, the infarct core area unexpectedly showed numerous blood vessels with a VD core of 29.46 ± 8.40% (Fig. 3G and H), which was 11.15 times greater than that on day 3 and 4.28 times greater than that on day 7. Moreover, the vessel density in the infarct core was significantly higher than that in the control cortex (19.85 ± 1.89%) and peri-infarct region (19.68 ± 1.91%) (Fig. 3I).

The microvessels in the infarct core were dysfunctional according to TEM (Fig. S1C), as the basement membrane was impaired or discontinuous and most vessel walls lacked mural features such as pericytes. Using immunofluorescence staining, we found that the number of proliferated endothelial cells labeled with BrdU and CD31 in the core region was also higher than that in the peri-infarct region (Fig. S1D). These findings provide strong evidence that increased angiogenesis in the infarct core is critical for functional recovery after stroke.

### Venogenesis rather than arteriosclerosis is the principal angiogenic event in the infarct core

**Fig. 4:**
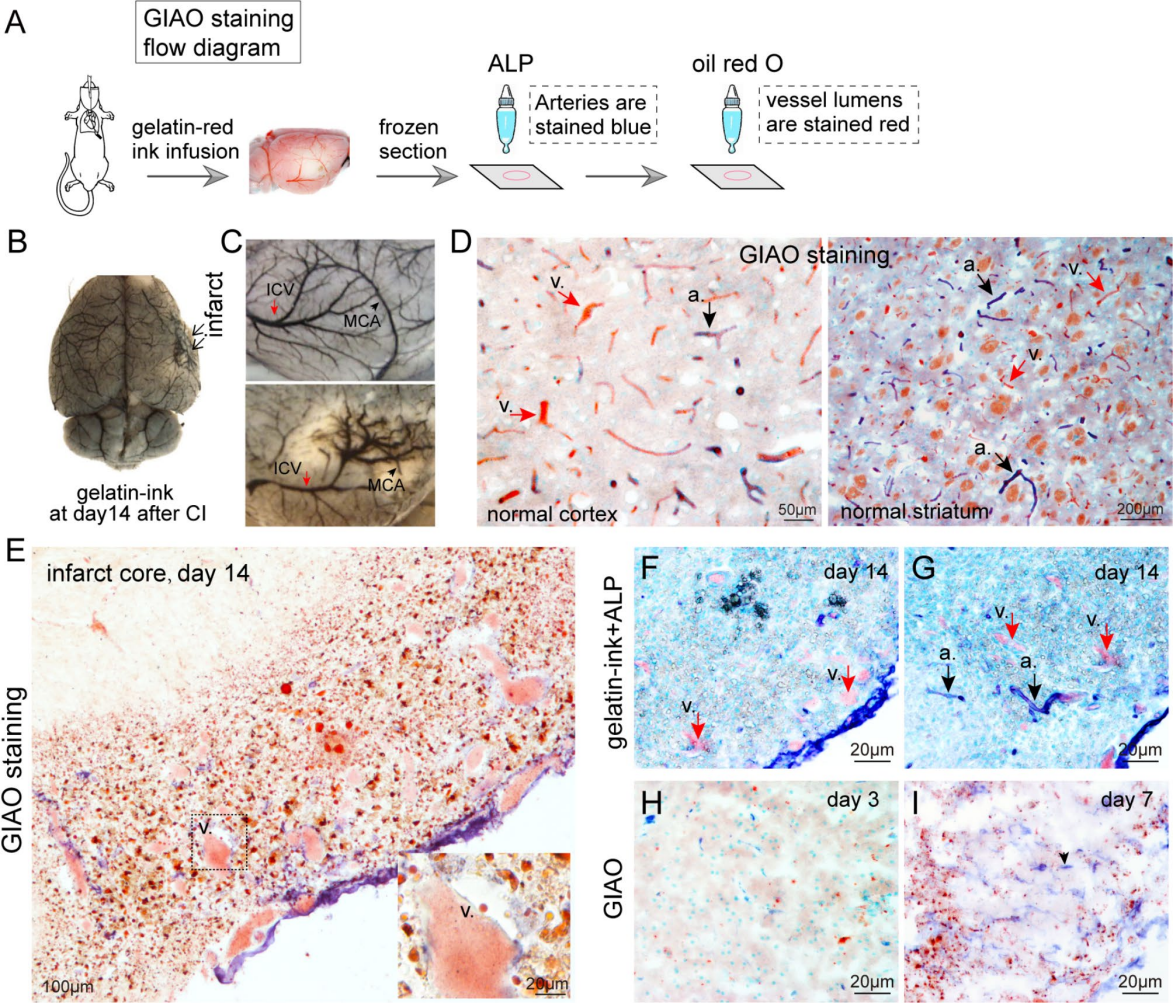
Venogenesis was the primary angiogenic event in the infarct core. **(A)** The flow diagram provides a concise overview of the GIAO staining procedures. **(B)** ~ **(C)** Gelatin-ink infusion for normal and ischemic brains on day 14. Red arrow, inferior cerebral vein (ICV); black arrow, middle cerebral artery (MCA). **(D)** GIAO staining was performed to distinguish arterial and venous capillaries in the brain. The arterial capillaries presented blue walls and red lumens. Only red lumens were visible in venous capillaries. Black arrow and *a.*, artery. Red arrow and *v.*, vein. **(E)** GIAO staining of the infarcted brain on day 14 after ischemia. The majority of newborn microvessels in the infarct core were veins, as ALP staining was either absent or weak in the majority of newborn microvessels. **(F)** and **(G)** Gelatin-ink + ALP staining of the infarct core. The arterial capillaries presented blue walls and red lumens. Venous capillaries presented with red lumens. Black arrow and *a.*, artery. Red arrow and *v.*, vein. **(H)** and **(I)** GIAO staining of the infarcted brain on days 3 and 7. None or few gelatin-ink labelled microvessels were present in the infarct area on days 3 and 7. However, endothelial progenitor cells (arrowheads) significantly proliferated in the infarct core on day 7

We defined venogenesis as the process of creating new veins, venules and venous segments of capillaries in brain tissue. To determine the phenotype of newborn vessels in the infarct core, we performed GIAO staining (Fig. 4A), which enabled us to clearly distinguish the arterial and venous segments of capillaries under light microscopy.

In gelatin-ink-infused brains, the inferior cerebral vein extended newly twisted and expanded branches into the infarct region on day 14 (Fig. 4B and C). Moreover, meaningeal venogenesis in the infarct core was associated with the absorption of necrotic tissue and functional recovery following stroke (Fig. S2). We then deduced that venogenesis rather than arteriogenesis was the predominant angiogenic event in the infarct core. In GIAO staining, the arterial capillary walls stained blue as a result of ALP activity, whereas the arterial lumen showed a red color due to the infusion of red ink, which was further emphasized by oil red O (Fig. 4D). The capillaries on the venous side appeared bright red only because of the absence of ALP (Fig. 4D). During the recovery period, the newly formed microvessels presented enlarged lumens and were distributed throughout the necrotic tissue (Fig. 4E-G). ALP activity was either absent or weak on the vascular walls at this time point (Fig. 4E-G); therefore, most of the newly formed microvessels in the infarct core were veins. As ischemia causes necrosis, no ALP-positive endothelial cells were observed in the infarct core in the acute phase (Fig. 4H). The proliferation of ALP-positive endothelial progenitor cells (EPCs) was observed in the infarct core one week later (Fig. 4I). However, determining the specific phenotype of endothelial cells on day 7 was difficult because they were immature and had not yet established new blood vessel tubes.

### Increased phagocytosis was associated with venogenesis in the infarct core during the recovery period

**Fig. 5:**
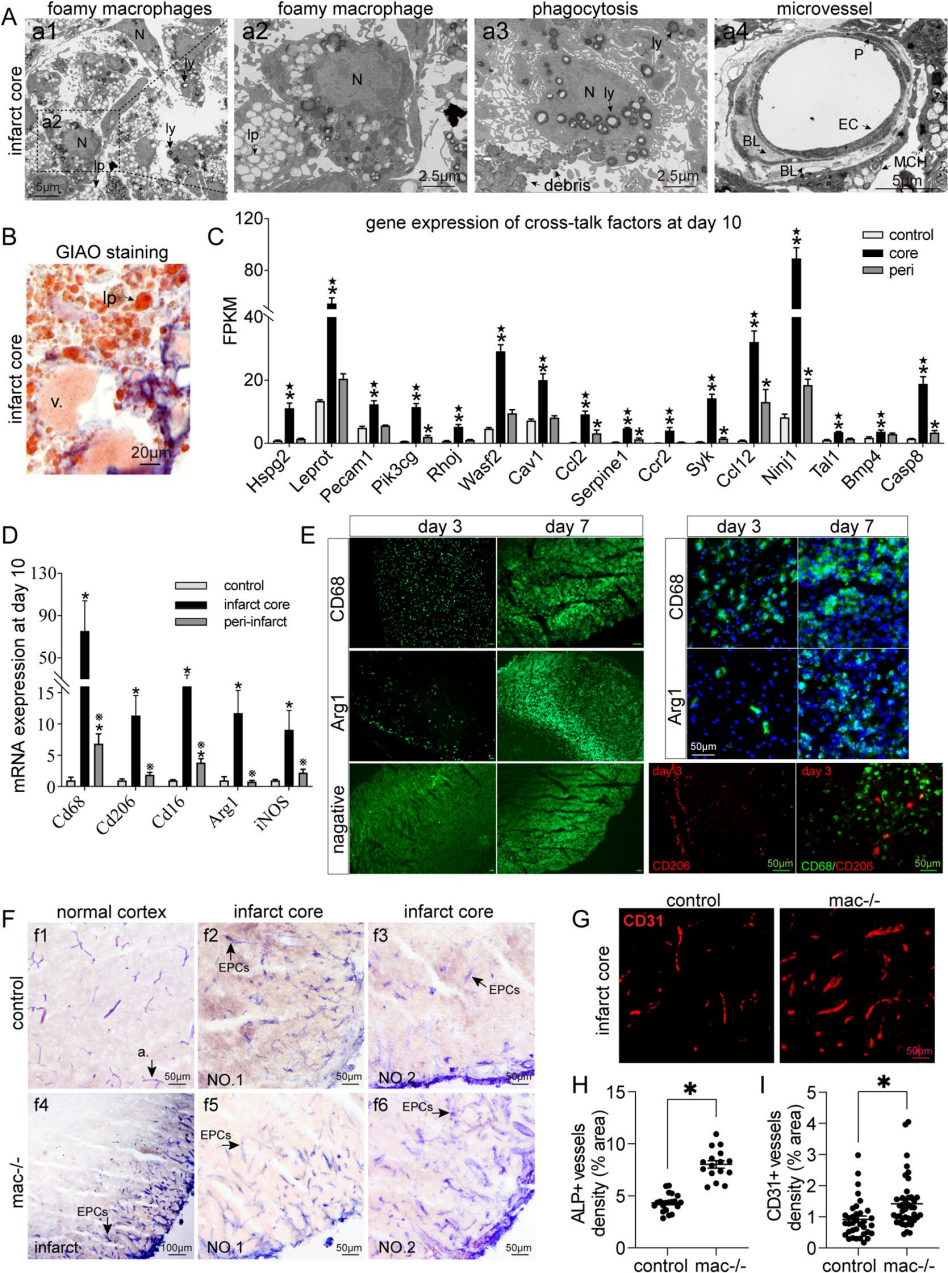
Phagocytosis was associated with venogenesis in the infarct core. **(A)** Transmission electron microscopy images of the infarct core on day 10. **(a1)** Foamy macrophages in the infarct region. **(a2)** Magnification of lipid-rich foamy macrophages. **(a3)** Macrophage engulf cell debris. **(a4)** A microvessel was spatially close to macrophages. ly, lysosome; lp, lipid droplets; N, nucleus; MCH, macrophage; ECs, endothelial cells; BL, basal lamina; P, pericytes. **(B)** GIAO staining revealed lipid-rich foamy macrophages and newly formed venules in the infarct core. **(C)** The top 20 upregulated phagocytosis-associated genes (fold change ≥ 2 and *p* < 0.05) in the infarct core (*n* = 5). **(D)** mRNA expression of macrophage markers of different phenotypes on day 10. *n* = 4. *p < 0.05 vs. control. ^※^*p* < 0.05 vs. peri. **(E)** Immunofluorescence images of CD68-, Arg1-, and Cd206-labeled macrophages in the infarct core on days 3 and 7. Scale bar, 50 μm. **(F)** ALP staining of microvessels and EPCs in the cortex. **(f1)** Arterioles in the normal cortex. **(f2) and (f3)** ALP-labeled EPCs in the infarct region of control mice. **(f4) ~ (f6)** ALP-labeled EPCs in mice with macrophage depletion (mac-/-). EPCs proliferate and form microvessels from the outer edge of the infarct cortex. **(G)** Representative CD31-labeled microvessels in the infarct cores of control and mac-/- mice. **(H)** and **(I)** Quantitative analysis revealed increased vessel density (ALP, *p* < 0.0001; CD31, *p* = 0.006) in mac-/- mice compared with control mice. *p < 0.05

In addition to angiogenesis, phagocytosis was also significantly increased in the infarct core during the recovery phase (Fig. 5). TEM and oil red O revealed abundant lipid-rich foamy macrophages in the infarct core on day 10 (Fig. 5A-B). RNA-seq revealed 284 and 261 upregulated DEGs, which regulate endocytosis and macrophage activation, in the infarct core compared with those in the healthy cortex and peri-infarct cortex, respectively (Fig. S3). These genes are predominantly involved in biological processes such as endocytosis, positive regulation of phagocytosis (such as Tyrobp, Trem2, and Apoe), macrophage activation, and macrophage chemotaxis and migration (Fig. S3A-C). Receptor-mediated endocytosis and phagocytosis are the primary mechanisms employed to remove necrotic tissue.

Lipid-rich macrophages were mostly distributed in the infarct core and infarct border zone, but few were present in the peri-infarct region and closely associated with newly formed veins (Figs. 4E and 5A-B). A total of 16 genes involved in cross-talk between angiogenesis and phagocytosis, such as Ccl2, Ccr2, Niji1 and Casp8, were increased in the infarct core (Fig. 5C; Fig. S3D).

During the recovery period, both the gene markers of M1 (CD16, iNOS, and CD68) and M2 (CD206 and Arg1) macrophages were elevated in the infarct center (Fig. 5D). In the acute phase (day 3), macrophage infiltration was primarily composed of the M1 type, while there was a slight increase in M2 type (CD206, Arg1) macrophages (Fig. 5E). By day 7, the number of M2-type (Arg1) macrophages had significantly increased (Fig. 5E), indicating a shift toward a more anti-inflammatory or phagocytotic response. This increase was accompanied by a notable increase in the number of EPCs (Fig. 5F). These observations indicate that increased phagocytosis within the infarct core may be associated with venogenesis and functional recovery during the recovery period. To further investigate the role of macrophages in venogenesis, clodronate liposomes (250 µL) were injected intraorbitally on the 4th to 5th day poststroke to deplete macrophages (mac-/-) in the circulation. By day 8, the CD31-labeled blood vessel density in the infarct core was significantly increased in mac-/- mice (Fig. 5G and I). ALP-positive progenitor endothelial cells in the infarct core also significantly increased after macrophage depletion (Fig. 5F and H), indicating that macrophage activation may inhibit venogenesis during the acute phase of stroke.

## Discussion

Angiogenesis is an important biological process in the infarct core during the recovery period after stroke. Vascular proliferation in the infarct core was noticeably greater than that in the peri-infarct region during the recovery period. Venogenesis rather than arteriogenesis was the main angiogenic event in the infarct core. Morphological and structural alterations in veins are important factors in the pathogenesis of ischemia and in the subsequent restoration of neuronal function following stroke.

Normalization of the morphology and functional remodeling of microvessels in the ischemic penumbra can aid in functional recovery after ischemic stroke [4, 5]. The transcription of angiogenesis-associated genes has been reported to be promoted soon after ischemia [15]. Endothelial cells proliferate in the injured area as early as 12–24 h after the onset of ischemia, and new blood vessel formation can continue for 21 days [16, 17]. The levels of proangiogenic factors, which are necessary for sprouting angiogenesis and tube formation and maturation, remain elevated in the peri-infarct region for 14 days after cerebral ischemia [18]. Our research, in contrast to previous findings, revealed that in addition to the peri-infarct zone, the infarct core also had increased vessel density on day 14. The expression of microvessel markers and vascular growth factors was increased in the peri-infarct region and infarct core during the recovery period after stroke. The upregulated angiogenesis-related genes in the infarct core are required for the regulation of endothelial cell proliferation and migration, reconstruction of the basal lamina, blood vessel morphogenesis, and other processes. Numerous molecules, including matrix metalloproteinases, cytokines, integrins, and growth factors, have been shown to promote angiogenesis. These elements support neuronal restoration by promoting angiogenesis, neurogenesis, synaptogenesis, and axonal plasticity through the activation of various signaling pathways. In this study, multiple signaling pathways involved in angiogenesis, such as AGE-RAGE, PI3K-Akt, apelin, HIF-1 and MAPK signaling, were found to be highly activated in the infarct core as well as in the peri-infarct region during stroke recovery. The expression of genes involved in these signaling pathways was noticeably greater in the infarct core than in the peri-infarct region, indicating strong vascular proliferation in the infarct core during the recovery stage after stroke.

Wei et al. [19] previously reported that modification of arterial CBF contributed to brain repair, as the arteriolar collaterals supplying the surface arterial network in the infarct region grew larger and more tortuous 30 days following ischemia. In the last two decades, however, only a few studies have proposed that there may be vascular proliferative activity in the infarct core due to the presence of proangiogenic factors and elevated levels of VEGF and BDNF in the infarct core [20, 21]. By using gelatin-ink infusion methods, we demonstrated that the infarct core displayed substantial vascular remodeling and had significantly more blood vessels than the peri-infarct region and healthy brain tissue during the recovery period. Angiogenesis involves a set of complex coordinated responses of signaling molecules and tissue components to adapt to the hypoxic microenvironment. The abundant proliferation and extension of endothelial cells in conjunction with the coordinated remodeling of the basal lamina matrix and the wrapping of pericytes contribute to angiogenesis in the peri-infarct region [4]. The microvessels gradually presented with an integrated structure and normal permeability of the blood–brain barrier during brain recovery. However, the newborn microvessels in the infarct core exhibited high permeability due to an abnormal vascular structure, particularly discontinuity of the basal lamina and encircling pericytes, indicating a heterogeneous phenotype of angiogenesis. A few studies have reported that leptomeningeal cells proliferate in response to ischemia and may be closely associated with vasculogenesis in the infarct region [22]. Nakagomi et al. reported that leptomeningeal cells presented neural stem cell activity and may develop into pericytes in the infarct area following ischemia [21, 22]. We also found that the proliferation and migration of meningeal cells may be the primary drivers of angiogenesis in the infarct core. The process of vascular proliferation typically starts at the outer edge of the infarct cortex and gradually progresses toward the center of the infarct. This progression occurred as new venous branches formed and expanded on the leptomeninges in an attempt to clear necrotic tissue after stroke. There may be complex interactions among meningeal cells, macrophages, and angiogenesis in the infarct core.

It has been reported that ischemia causes transient angiogenesis and microvessel leakage at the inner edge of the cystic infarct area, and the vascular hyperdensity decreases along with macrophage invasion 30 days later [23]. In this study, the majority of the new microvessels in the infarct core were veins because of the absence of ALP on proliferative endothelial cells. More direct evidence for venogenesis is that the neonatal leptomeningeal vessels in the infarct cortex mainly originate from the proliferating branches of the inferior cerebral veins. In contrast to the increased microvessel density in the peri-infarct area, venogenesis in the infarct core was temporary and retracted simultaneously with the absorption of necrotic tissue. Jugular vein ligation during the recovery period delayed the restoration of neuronal function in the present study. These findings confirmed our hypothesis that venogenesis in conjunction with macrophage infiltration in the infarct core promotes necrotic tissue absorption and is associated with better long-term functional recovery following cerebral infarction.

The cerebral venous system controls blood flow out of the brain and is crucial for regulating cerebrovascular events [2]. Venous drainage may be associated with the arterial collateral and infarct volumes in patients with stroke due to proximal artery occlusion [24]. Favorable venous outflow reflects excellent tissue perfusion, predicts good functional outcomes in patients with acute ischemic stroke due to large vessel occlusion after thrombectomy treatment [25, 26] and is independently associated with successful and excellent first-pass reperfusion [3]. Interruption of ipsilateral venous outflow in the acute period caused an increased infarct volume and a poorer outcome in the present study. The larger terminals, more tortuous branches, and enhanced CBF of the draining veins reflected increased venous drainage, which may have successfully reduced cerebral edema in the acute stage after stroke.

Macrophage infiltration facilitates neurovascular remodeling because cerebral ischemia initiates significant genomic reprogramming of macrophages that invade the brain [27]. The proliferation and activation of border-associated macrophages in the meningeal perivascular space [28] and the infiltration of monocyte-derived macrophages into the infarct border region in the acute phase may play pivotal roles in regulating venogenesis in the infarct core [29]. In the acute phase of stroke, the activation of border-associated macrophages may inhibit venogenesis, as evidenced by the fact that macrophage depletion in the acute phase increases the number of new blood vessels in the infarct core. This may be because the unique proinflammatory activity of monocytes/macrophages identified by transcriptomic profiles impairs the brain parenchyma in ischemic lesions [30]. However, macrophages in the infarct core during the recovery period exhibited a marked phagocytic phenotype with foam transformation due to lipid digestion and may be closely associated with increased venogenesis in the infarct core. However, macrophages adjacent to the healthy cortex in the ischemic region lack phagocytic morphology. In agreement with our observations, Manoonkitiwongsa et al. reported that macrophage infiltration primarily occurred in necrotic areas as opposed to the penumbra [31]. Phagocytosis of dead cells and clearance of cell debris are crucial steps in the brain’s repair process following a stroke. We detected an increase in the expression of numerous genes related to phagocytosis in the infarct core during the recovery process. These genes include find-me molecules such as Cx3cr1 and Lrp1, as well as eat-me molecules such as Axl and Mertk. Additionally, we also observed an upregulation of cross-talk genes that are associated with the regulation of angiogenesis and macrophages. Further research is needed to fully understand the mechanisms underlying this process and explore potential therapeutic interventions aimed at promoting angiogenesis or necrotic absorption after stroke.

In this study, inflammation and the immune system played predominant roles in peri-infarct and necrotic tissue during the recovery period, indicating that the inflammatory response may contribute to promoting vascular remodeling during recovery. By triggering intrinsic signaling pathways, inflammatory cells release numerous cytokines that influence endothelial cell proliferation, migration, and activation, contributing to the angiogenesis process [32]. However, the mechanism of venogenesis in the infarct core is still not fully understood and requires further research.

In summary, cerebral infarction results in substantial angiogenesis in both the central and surrounding areas of ischemia. Venogenesis was the primary angiogenic event in the infarct core throughout the recovery period after stroke. Favorable venous outflow helps alleviate inchemic injury in the acute phase of cerebral infarction. Venogenesis, which occurs in the infarct core area during the recovery stage, is a transient process aimed at promoting the absorption of necrotic tissue after stroke. The activation and infiltration of macrophages in the acute phase of stroke inhibits venogenesis in the infarct core. However, macrophages may promote venogenesis during the recovery stage and contribute to functional recovery by actively removing necrotic cell debris in the infarct area.

## Electronic supplementary material

Below is the link to the electronic supplementary material.

Supplementary Material 1

Supplementary Material 2

Supplementary Material 3

Supplementary Material 4

## Data Availability

The data will be made available upon reasonable request.

## Abbreviations

CBF: Cerebral blood flow
MCA: Middle cerebral artery
TTC: 2,3,5-triphenyltetrazolium chloride
MCAO: Middle cerebral artery occlusion
GIAO: Gelatin-ink-alkaline phosphatase-oil red
ALP: Alkaline phosphatase
TEM: Transmission electron microscopy
LSCI: Laser speckle contrast imaging
RNA-seq: RNA sequencing
GO: Gene Ontology
24 h-LJV: Ligation of the right jugular vein at 24 h
7d-LJV: Ligation of the right jugular vein at 7 days
PCA: Principal component analysis
DEGs: Differentially expressed genes
VD-peri: Peri-infarct vessel density
EPC: endothelial progenitor cells
