# Supplementary material for: Key mechanisms of angiogenesis in the infarct core: association of macrophage infiltration with venogenesis
Source: Mol Brain. 2025 Feb 14;18:12. doi: 10.1186/s13041-025-01182-1 (PMC11827325; doi:10.1186/s13041-025-01182-1)
Supplement: Supplementary file 1 — Supplementary Material 1 [file 13041_2025_1182_MOESM1_ESM.docx]

**Supplementary figure legends**

Fig. S1 Angiogenic activity was increased during recovery period. (A) qPCR analysis showed increased mRNA expression of angiogenic factors in infarct core at day 10 after ischemia. *p < 0.05, infarct core vs. control. ※p < 0.05, infarct core vs. peri-infarct. **(B)** GO analysis of the upregulated angiogenesis-related DEGs showed the top enriched items that were involved in vascularization process in the infarct core on day 10. G1, comparison between normal control cortex and infarct core. G2, comparison between peri-infarct cortex and infarct core. **(C)** Transmission electron microscopy images. Normal endothelial cells (ECs) were enveloped by the basal lamina (BL) and pericytes (P). N, nucleus. Microvessel in the infarct core were missing a mature wrapping structure and disintegrated BL. **(D)** Colocalization of CD31/BrdU showing strong proliferative activity on large and immature microvessels in the infarct core.

**Fig. S2 Venogenesis in the infarct core was associated with absorption of necrotic tissue during the recovery period.** **(A)** Gelatin ink-infused brains showing venogenesis in the infarct core after cerebral infarction. **(B)** Gelatin-ink labelled brain sections showed that increased venogenesis was associated with clearance of necrotic tissue. **(C)** Cerebral cortex gradually shrink one week later after stroke. *p < 0.05 vs. day 3. ^※^p < 0.05 vs. day 7. **(D)** The Pearson correlation coefficient showed statistically negative correlation between blood vessel density and infarct area in infarct core region. **(F)** The line chart indicated improvement in motor function as the microvessel density continued to increase over the course of two weeks.

Fig. S3 **Increased phagocytosis in the infarct core. (A) and (B)** GO analysis of the upregulated DEGs (fold change ≥2 and p < 0.05) revealed the items that were associated with phagocytosis and macrophage activity in the infarct core on day 10. **(C)** The top 20 upregulated phagocytosis-associated genes (fold change ≥2 and p < 0.05) in the infarct core. **(D)** Gene expressions of cross-talk factors between angiogenesis and phagocytosis. *p < 0.05 vs. core. ^★^p < 0.05 vs. peri.
