# Supplementary figures and images for: Key mechanisms of angiogenesis in the infarct core: association of macrophage infiltration with venogenesis
Source: Mol Brain. 2025 Feb 14;18:12. doi: 10.1186/s13041-025-01182-1 (PMC11827325; doi:10.1186/s13041-025-01182-1)

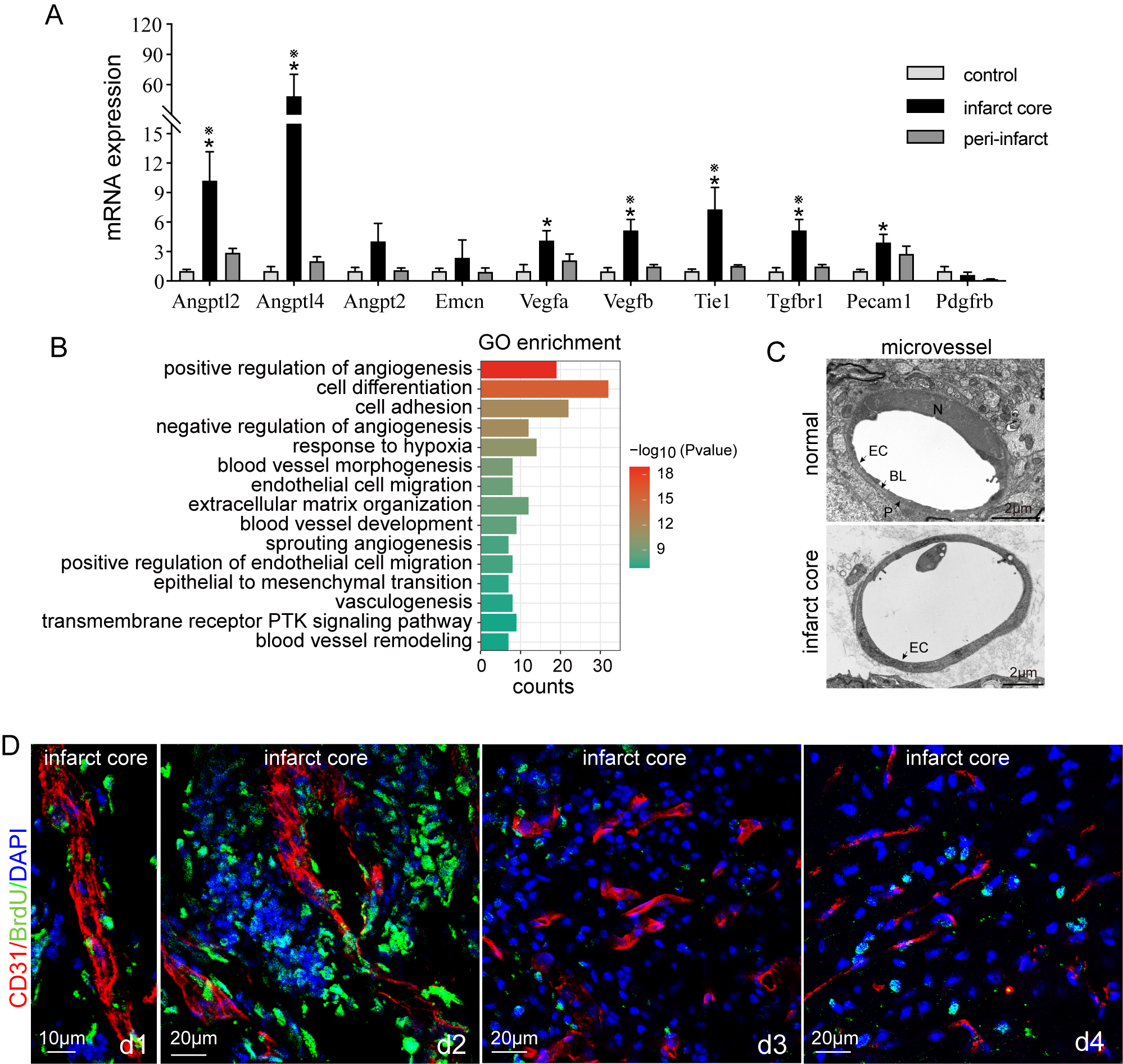

Supplement: Supplementary file 2 — Supplementary Material 2 [file 13041_2025_1182_MOESM2_ESM.tif]

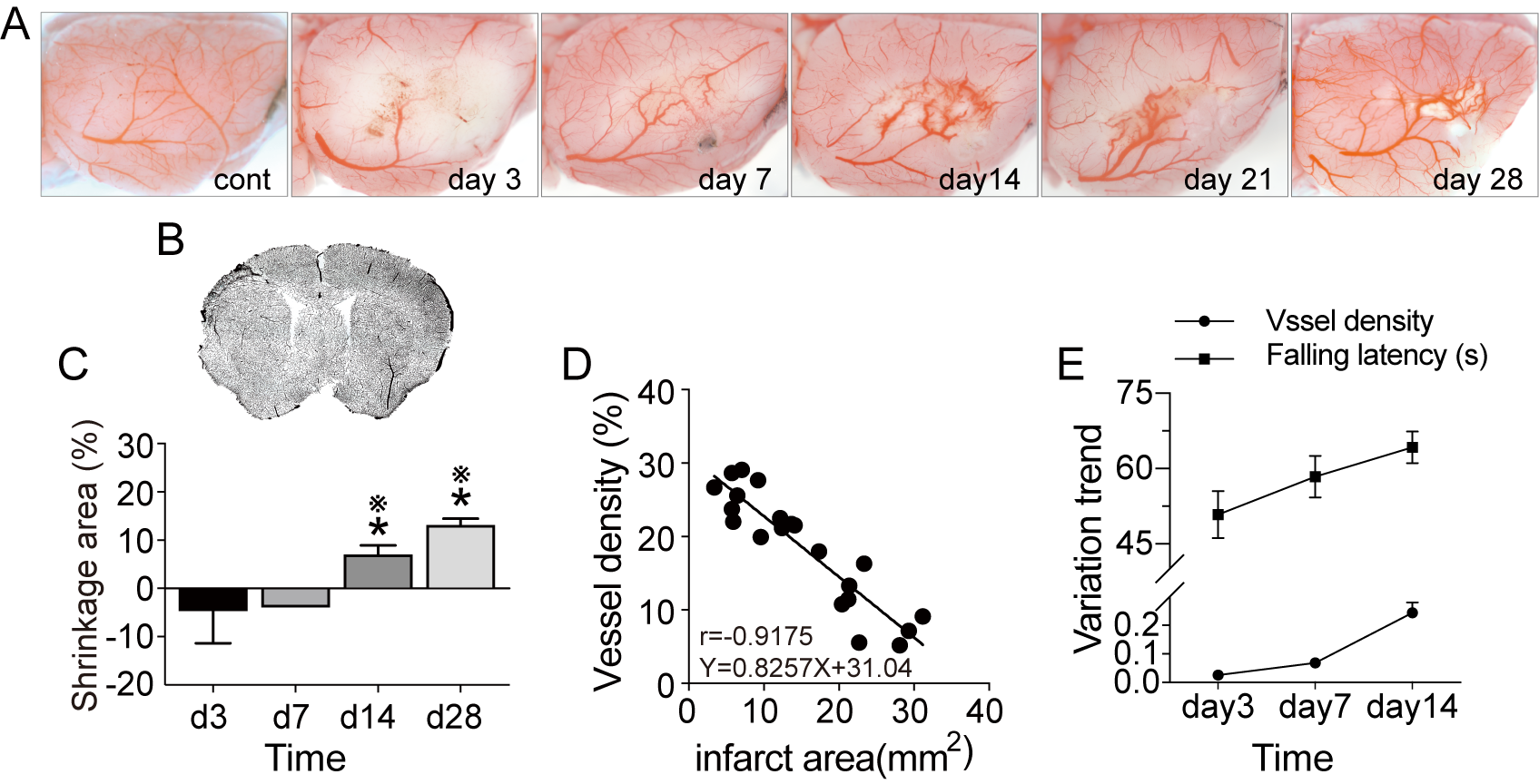

Supplement: Supplementary file 3 — Supplementary Material 3 [file 13041_2025_1182_MOESM3_ESM.tif]

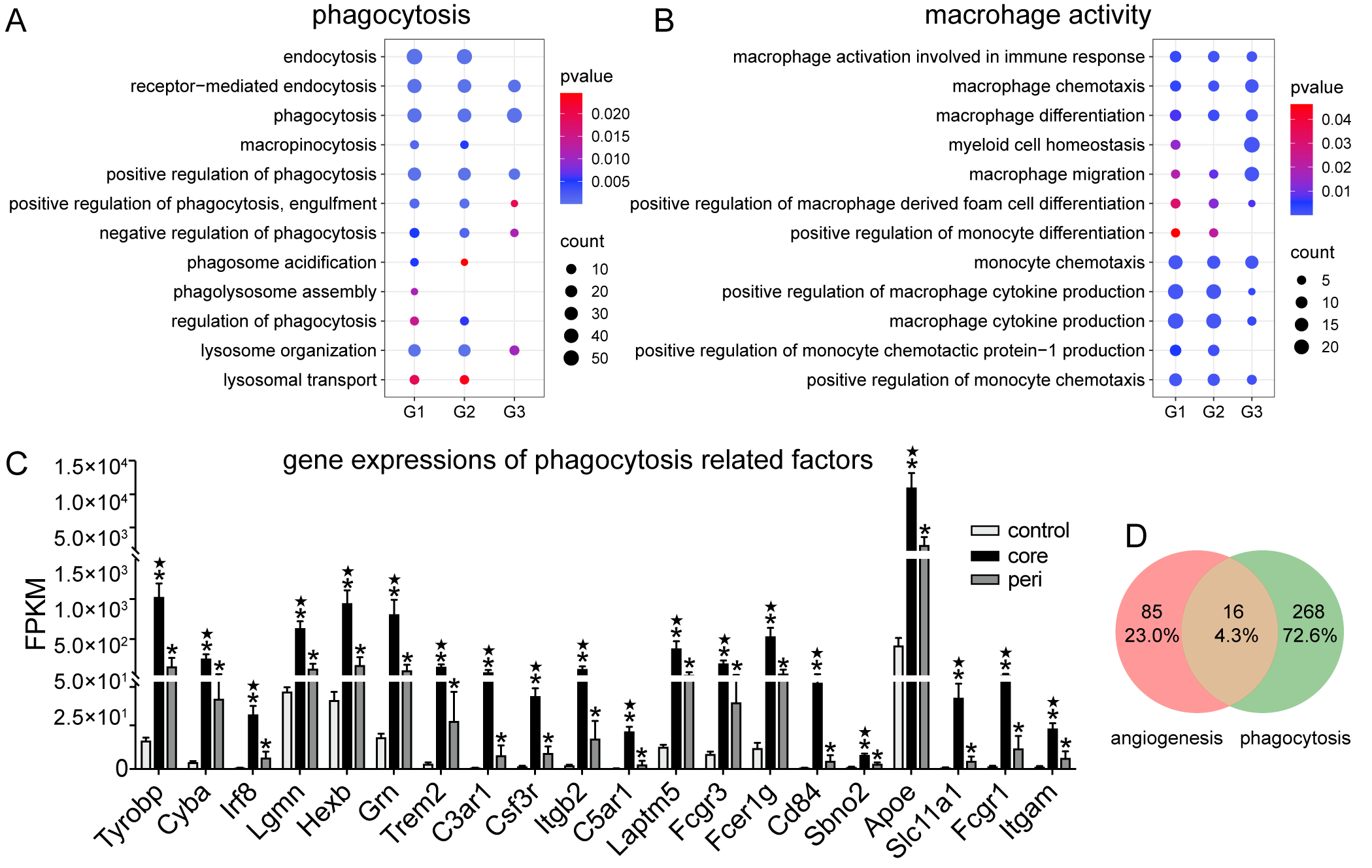

Supplement: Supplementary file 4 — Supplementary Material 4 [file 13041_2025_1182_MOESM4_ESM.tif]
